# Dilated cardiomyopathies and non-compaction cardiomyopathy

**DOI:** 10.1007/s00059-020-04903-5

**Published:** 2020-02-27

**Authors:** A. Hänselmann, C. Veltmann, J. Bauersachs, D. Berliner

**Affiliations:** Dept. of Cardiology and Angiology, Hannover Medical School, Carl-Neuberg-Str. 1, 30625 Hannover, Germany

**Keywords:** Heart failure, Myocarditis, Arrhythmias, cardiac, Peripartum cardiomyopathy, Genetics, Herzinsuffizienz, Myokarditis, Arrhythmien, kardiale, Peripartale Kardiomyopathie, Genetik

## Abstract

Dilated cardiomyopathy (DCM) is the most common form of cardiomyopathy and one of the most common causes of heart failure. It is characterized by left or biventricular dilation and a reduced systolic function. The causes are manifold and range from myocarditis to alcohol and other toxins, to rheumatological, endocrinological, and metabolic diseases. Peripartum cardiomyopathy is a special form that occurs at the end of or shortly after pregnancy. Genetic mutations can be detected in approximately 30–50% of DCM patients. Owing to the growing possibilities of genetic diagnostics, increasingly more triggering variants and hereditary mechanisms emerge. This is particularly important with regard to risk stratification for patients with variants with an increased risk of arrhythmias. Patient prognosis is determined by the occurrence of heart failure and arrhythmias. In addition to the treatment of the underlying disease or the elimination of triggering harmful toxins, therapy consists in guideline-directed heart failure treatment including drug and device therapy.

## Abstract (de)

Die dilatative Kardiomyopathie (DCM) ist die häufigste Form der Kardiomyopathie und eine der häufigsten Ursachen einer Herzinsuffizienz. Gekennzeichnet ist sie durch eine links- oder biventrikuläre Dilatation sowie eine reduzierte systolische Funktion. Die Ursachen sind mannigfaltig und reichen von einer Myokarditis über Alkohol und andere Toxine bis hin zu rheumatologischen, endokrinologischen und metabolischen Erkrankungen. Eine Sonderform stellt die peripartale Kardiomyopathie dar, die gegen Ende oder kurz nach der Schwangerschaft auftritt. Bei etwa 30–50 % der von einer DCM betroffenen Patienten können genetische Mutationen nachgewiesen werden. Durch die wachsenden Möglichkeiten der genetischen Diagnostik werden immer mehr auslösende Varianten und Vererbungsmechanismen bekannt. Dies hat Bedeutung v. a. für die Risikostratifizierung bei Varianten mit erhöhtem Risiko für Arrhythmien. Die Prognose der Patienten wird durch das Auftreten der Herzinsuffizienz und von Arrhythmien bestimmt. Neben der Behandlung der Grunderkrankung oder dem Verzicht auf auslösende Noxen sind eine leitliniengerechte medikamentöse und Devicetherapie der Herzinsuffizienz erforderlich.

Cardiomyopathies represent an inhomogeneous group of cardiac diseases with structural and functional changes in the myocardium that can cause heart failure and death [1, 2]. During the past few years a better characterization of myocardial diseases became possible through more accurate techniques and genetic diagnostics.

In 1980, the World Health Organization (WHO) defined cardiomyopathies as myocardial diseases of unknown cause to distinguish cardiomyopathies from myocardial diseases secondary to hypertension, coronary heart disease, or valvular disease [2]. In 1996, this classification was extended to all diseases of the heart muscle—the WHO defined cardiomyopathies as myocardial diseases associated with cardiac dysfunction [3]. The current definition of the European Society of Cardiology (ESC) defines cardiomyopathies as myocardial dysfunction in which the myocardium is structurally and functionally altered in the absence of coronary heart disease, hypertension, valvular disease, or congenital heart disease [4].

The current definition of cardiomyopathies is based on intense research and divides the disease according to its morphology, with each phenotype also being divided into a familial and nonfamilial form [5]. The symptoms of patients can vary greatly, even in members within one family. Patients with dilated cardiomyopathy (DCM) can develop reduced ejection fraction and, as the disease progresses, an increased risk of arrhythmias and sudden cardiac death [1].

## Definition of dilated cardiomyopathy

Dilated cardiomyopathy has been defined as dilatation of the left or both ventricles that is not explained by abnormal loading conditions (e.g., hypertension, valvular diseases) or coronary artery disease sufficient to cause global systolic impairment [4]. In 2016 a revision of the definition of DCM introduced a new category of so-called hypokinetic non-dilated cardiomyopathy (HNDC) in addition to the classic definition of DCM [6]. Hypokinetic non-dilated cardiomyopathy was defined as left ventricular or biventricular global systolic dysfunction without dilation (defined as left ventricular ejection fraction [LVEF] <45%), which is not explained by abnormal loading conditions or coronary heart disease [6].

The guidelines of the American Heart Association (AHA) emphasize that the distinction between ischemic and non-ischemic cardiomyopathy is frequently used in everyday clinical practice. The term “non-ischemic cardiomyopathy” is often used in a similar way to the term DCM. However, it is neglected that the former term also includes the forms of cardiomyopathies that are due to volume or pressure load and by definition are not classified under the DCM [7].

## Epidemiology and prognosis of dilated cardiomyopathy

Dilated cardiomyopathy is the most common form of cardiomyopathy while its exact prevalence is unknown. In the general population, a prevalence of approximately 1:250 is assumed, of which 30–50% of cases are genetically determined [5, 8]. Dilated cardiomyopathy is the most common cause of heart failure in young patients. Thereby, it represents the most frequent etiology for patients undergoing heart transplantation [5]. Including pediatric patients, the prevalence of non-ischemic cardiomyopathy is higher than that of ischemic cardiomyopathy [7]. It is believed that DCM is the underlying cause of heart failure in approximately one third of cases [9]. Dilated cardiomyopathy can occur at any age, but in most cases it manifests in the third or fourth decade of life. Age is a risk factor for mortality in patients with DCM [7]. The prognosis is mainly determined by the onset and course of heart failure. Approximately 25% of patients with DCM with a new onset of symptoms show a spontaneous improvement. Patients with symptoms persisting for more than 3 months with initial cardiac decompensation have a significantly worse chance of recovering [10]. Patients with idiopathic DCM appear to have a better prognosis than patients with other forms of DCM [11]. The causes of death are attributed in about one-third to sudden cardiac death, and in two-thirds to a progressive pump failure. Overall the prognosis seems to be better in DCM than in ischemic cardiomyopathy [7].

## Diagnosing dilated cardiomyopathy

### Clinical presentation

**Fig. 1:**
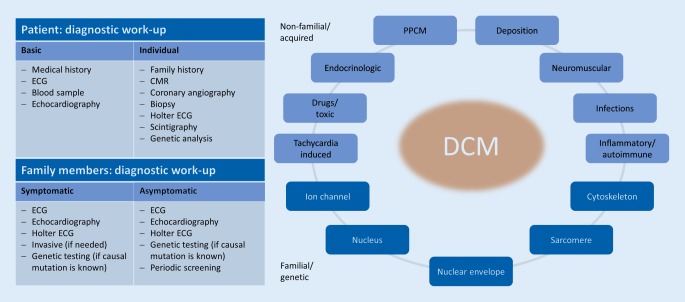
Causes of dilated cardiomyopathy (*DCM*): a diagnostic work-up. *CMR* cardiovascular magnetic resonance imaging, *ECG* electrocardiography, *PPCM* peripartum cardiomyopathy. (Modified from [6–9, 51, 61, 62])

Dilated cardiomyopathy can become clinically apparent in different ways: in 75–85% of patients with signs and symptoms of heart failure, in 86% dyspnea on exertion, in 30% palpitations, in 29% peripheral edema. During the course of the disease, 95% of patients develop symptoms of heart failure. In 4–13% of patients, asymptomatic cardiomegaly is detected in the beginning [12]. Other forms of initial presentation include coexisting arrhythmia, conduction disturbance, thromboembolic complications, or sudden death ([13]; Fig. 1).

### The significance of imaging

Echocardiography is the most important method, showing left ventricular dilatation, which can reach massive proportions and thus lead to an increase in total cardiac muscle mass. Owing to the structural dilatation, the geometry is more like a sphere than an ovoid. The wall thickness itself is normal or reduced. The left ventricular end-diastolic volume index (EDVI) is often >100 ml/m^2^ (normal <75 ml/m^2^). Dilated cardiomyopathy is associated with a decrease in systolic indices (i.e., LVEF; [4]). In addition to isolated left ventricular dilatation, enlargement of all four heart cavities can be observed. Secondary mitral regurgitation is common [14].

Beside systolic dysfunction, diastolic dysfunction can also be observed. The occurrence of advanced forms of diastolic dysfunction (i.e., restrictive or pseudonormal filling patterns) has been associated with a poorer prognosis [15].

Cardiac magnetic resonance (CMR) is recognized as the gold standard for measuring the volume, mass, and ejection fraction of both ventricles [16]. The key advantage of CMR is the possibility of tissue characterization. T1 and T2 mapping allow for a more accurate assignment of the underlying pathology of DCM. Myocardial fibrosis, edema, inflammation, and infiltrative disorders lead to changes in T1 relaxation time. Longer T2 relaxation times are caused by myocardial edema and indicate myocarditis, stress cardiomyopathy, and sarcoidosis. The detection of fibrosis is possible with the help of late gadolinium enhancement (LGE). Depending on the distribution pattern, LGE allows for the differentiation between ischemic and non-ischemic etiologies. Mid-wall LGE in non-ischemic cardiomyopathy is frequent (approximately one third of DCM patients). It reflects fibrosis, which has been shown to be a strong and independent predictor of all-cause mortality, cardiovascular death/transplantation, and sudden cardiac death ([17, 18]; Fig. 1).

### Endomyocardial biopsy

Endomyocardial biopsy (EMB) continues to be the gold standard in the detection of myocardial inflammation and fibrosis, even though invasiveness and so-called sampling error detract from its use. Nevertheless, biopsy findings offer clear therapeutic recommendations, especially if giant cell myocarditis or sarcoidosis is diagnosed ([19, 20]; Fig. 1).

### Excluding other causes of left ventricular dilatation

The most important differential diagnosis to be excluded is ischemic cardiomyopathy (ICM). Although not truly a cardiomyopathy by definition, this term is commonly used. Clinical and echocardiographic pictures of ICM and DCM can be very similar. Previously, ICM was defined by the following: [21]

- Patients with history of myocardial infarction (MI) or revascularization (coronary artery bypass grafting [CABG] or percutaneous coronary intervention [PCI])
- Patients with 75% stenosis of the left main or proximal left anterior descending artery (LAD)
- Patients with 75% stenosis of two or more epicardial vessels

Valvular heart disease should be ruled out, but it should be noted that a sustained DCM due to dilatation is often associated with a higher grade of secondary mitral regurgitation [22].

## Etiology of dilated cardiomyopathy

The causes for DCM are manifold and can be classified as familial, i.e., genetic as well as non-familial (acquired) forms [4]. Infections, drugs toxic substances, or autoimmune diseases can cause the appearance of a DCM leading to a complex disease with many overlapping environmental and genetic factors (Table 1; [23]).

**Table 1:** Overview of the most common diseases or agents leading to DCM (modified from [6, 7, 60])

Disease or agent	Comments
*Infections*	
Viral	Adenovirus, HIV, Coxsackie, Cytomegalovirus, Varicella, Epstein–Barr viruses
Bacterial	Streptococci, Diphtheria, Typhoid fever, Mycobacteria, Brucellosis
Fungal	Histoplasmosis, cryptococcosis
Parasitic	Toxoplasmosis, trypanosomiasis, schistosomiasis, trichinosis
Spirochetal	Leptospirosis, syphilis, Lyme disease
*Toxic*	
Ethanol (alcoholic cardiomyopathy)	Biventricular dysfunction and dilatation, absence of other causes → abstinence; often good response after withdrawal
Cocaine	Long-term use, direct toxicity for the myocardium
Methamphetamine	Fourfold increased risk of developing cardiomyopathy → in the case of abstinence, recovery is possible
*Deposition*	
Amyloidosis	Starts with restrictive cardiomyopathy, can progress to severe systolic dysfunction → amyloid identification and treatment thereafter
Iron	Hemochromatosis
*Neuromuscular*	
Duchenne muscular dystrophy; Becker muscular dystrophy	X‑linked; CK elevation; family screening
*Drugs*	
Chemotherapeutic Agents	Anthracyclines (early and late events), cyclophosphamide, trastuzumab
Psychiatric drugs	For example, lithium, clozapine, risperidone, tricyclic antidepressants → stop therapy; in the case of recovery, re-initiation can be discussed
*Autoimmune/Inflammatory*	
Giant cell myocarditis	Multinucleated giant cell; AV block, VT
Inflammatory DCM	Non-infectious myocarditis, in biopsy
Lupus erythematosus, Polymyositis, Wegner’s granulomatosis, Churg–Strauss syndrome, rheumatoid arthritis, dermatomyositis	Development of DCM possible but rather scarce; biopsy → treatment depends on underlying cause
Myocarditis	Any inflammatory disease of the myocardium, biopsy, causes vary
Sarcoidosis	Granulomatous inflammation, abnormal systolic and diastolic function, DCM is possible; high risk for arrhythmias → corticosteroids, immunosuppressive therapy (azathioprine), ICD
*Endocrinologic*	
Hypo- and hyperthyroidism	–
Cushing disease/Addison disease	–
Pheochromocytoma	–
Acromegaly	–
Diabetes mellitus	–
*Other*	
Peripartum cardiomyopathy	Last month before birth or in the first few months thereafter, predisposing factors: hypertension, Black race, age >30 years, multiparity, pre-eclampsia, smoking, family history and diabetes → with unstable hemodynamics urgent delivery
Tachycardia-induced cardiomyopathy	Caused by increased ventricular rate; arrhythmias; RV pacing → rhythm or frequency control, catheter ablation; CRT (D)
Electrolyte/renal	Hypocalcemia, hypophosphatemia, uremia
Nutritional deficiencies	Thiamine, selenium, carnitine, niacin

*DCM* dilated cardiomyopathy, *HIV* human immunodeficiency virus, *CMR* cardiac magnetic resonance, *CK* creatine kinase, *AV* atrioventricular, *RV* right ventricular, *VT* ventricular tachycardia, *ICD* implantable cardioverter-defibrillator, *CRT(D)* cardiac resynchronization therapy (with defibrillator)

DCM is often the final stage of various underlying diseases, whereby the diagnosis thereof offers specific therapeutic options (e.g., myocarditis, hemochromatosis). Another challenge is the growing number of patients who develop DCM/heart failure after drug therapy (e.g., chemotherapeutics; [24]).

Typical causes leading to DCM are presented in the following (and in Table 1). Different entities occurring simultaneously may amplify each other regarding the resulting DCM phenotype (e.g., genetic cause + excessive alcohol consumption; Fig. 1).

### Inflammation

Viral infection is the most common cause of myocarditis but also idiopathic-inflammatory or autoimmune-mediated cardiomyocyte destruction may induce an inflammatory process. Inflammation is either mediated via direct viropathic effects or indirectly via T‑cell-mediated immune processes [25]. Besides viruses, fungi, parasites, and chemotoxines may lead to inflammation. The progression to DCM probably evolves over months. Further causes of myocarditis are autoimmune or immune-mediated disorders (e.g., systemic lupus erythematosus, Wegener’s granulomatosis, giant cell arteritis, and Takayasu arteritis; [19]). Cardiac autoantibodies to various cardiac and muscle-specific autoantigens are found in myocarditis and in DCM patients but the role of autoantibodies in the progression from myocarditis to DCM is still not fully understood [19].

### Drugs, cardiotoxins, and chemotherapeutic agents

A number of chemicals can trigger DCM (Table 1). Among these, chronic alcohol consumption is the most common cause (21–32% of DCM patients; [6]). Alcohol enhances diastolic blood pressure, increases heart rate, and reduces myocardial perfusion. Moreover, ethanol and its metabolites directly cause structural and functional changes in the myocardium and increase myocyte loss due to apoptosis. The pathogenetic process leading to alcoholic cardiomyopathy is not fully understood. Currently, it is assumed that chronic alcohol consumption leads to an impairment of myocardial function due to toxic effects on myocyte sarcoplasm, mitochondrial dysfunction, oxidative stress, and impaired calcium homeostasis [26].

The creation of potentially cytotoxic reactive oxygen species, alterations in the topoisomerase IIβ pathway, and genetic predisposition are the proposed causes for the cardiotoxic effects of anthracyclines. There are other chemotherapeutic agents that can trigger cardiomyopathy (e.g., trastuzumab, certain antivascular endothelial growth factor inhibitors, and several proteasome inhibitors; [7]). The recently introduced cardiologic subspecialty “cardio-oncology” deals with the cardiotoxic effects of such cancer therapies.

### Peripartum cardiomyopathy

Another entity that also leads to the clinical appearance of a DCM is so-called peripartum cardiomyopathy (PPCM). Initially described in 1849, this entity is still underdiagnosed.

Peripartum cardiomyopathy is the most common form of cardiomyopathy in pregnancy and usually occurs in the last month before birth or in the first few months thereafter [27]. The defining criteria of PPCM are heart failure with reduced LVEF (<45%) and the absence of other causes for heart failure [28]. The symptoms often correspond to those of heart failure, but the phenotype is variable. The manifestation can occur with mild symptoms but also with very acute onset up to cardiac arrest [29]. Other causes of heart failure and other pre-existing cardiomyopathies have to be ruled out. The overlap with pregnancy-related symptoms complicates the diagnosis and is the main reason that the PPCM is still underdiagnosed [28].

Predisposing factors for PPCM are hypertension, African, age >30 years, multiparity, pre-eclampsia, smoking, family history, and diabetes. The incidence of PPCM ranges widely, the highest reported incidence is 1:100 in Nigeria, while it is 1:1500 in Germany [28, 30].

The exact pathogenesis remains unclear; a possible mechanism is the production of a 16-kDa fragment of the nursing hormone prolactin leading to endothelial and cardiomyocyte damage [31]. Inhibition of prolactin release by the dopamine D2 receptor agonist bromocriptine is a potential pathophysiologically based therapeutic option [32].

There are overlaps between PPCM and DCM. It was observed that in about 15–20% of patients with PPCM there were genetic variants, e.g., lamin A/C, myosin-binding protein C, titin, and others associated with cardiomyopathies [28, 33]. In these patients a DCM may be unmasked by pregnancy. Genetic testing in PPCM patients may therefore be considered in the case of a positive family history [28]. Registry comparisons have revealed indications of a genetic link between PPCM and the occurrence of cancer. Patients with a history of chemo-/radiotherapy for malignant disease should be screened via echocardiography before and during pregnancy [34].

Echocardiography and biomarkers for heart failure are important for diagnosing PPCM. During pregnancy, slightly elevated values of B‑type natriuretic peptide (BNP) and NT-proBNP are possible, but in the case of acute PPCM they can be seen regularly; so far, there is no specific biomarker for the diagnosis of PPCM [28].

## Genetics of DCM

The detailed work-up of the patient’s history including a comprehensive medical history of the family (e.g., heart failure, sudden cardiac death, transplantation, pacemaker, or stroke in younger age) is decisive to estimate the probability of a genetic origin of the cardiomyopathy [35]. If two or more sick relatives are found in a family, a familial DCM can be assumed [36]. In this case, genetic testing should be considered. Genetic testing has several possible indications [10]:

- Identification of etiology
- Risk assessment (e.g., recommended implantation of an implantable cardioverter-defibrillator [ICD] in certain gene variants)
- Predictive testing of relatives
- Advice on family planning.

More than 40 genes are known today and mostly code for ion channels, sarcomeres, Z‑discs, nuclear proteins, and desmosomes (Table 2; [8, 36]). Today, many genes may overlap with other diseases or forms of cardiomyopathy. The overlaps with arrhythmogenic cardiomyopathies are particularly noteworthy here. For example, an LMNA mutation is associated with an increased risk of ventricular arrhythmia, regardless of the severity of the limitation of left ventricular function or degree of dilatation [37]. Muscular dystrophies also show a large overlap, so that such patients should also be examined for the presence of cardiomyopathy [8]. Inheritance in DCM is usually autosomal dominant with variable penetrance. However, X‑linked, mitochondrial, or recessive inheritance can also be found. Many genetic mutations can be found and the interpretation and classification of these mutations can be very challenging [8]. Some of the most common genetic aberrations affect the genes listed in Table 2.

**Table 2:** Overview of the most common known genes as a cause of familial dilated cardiomyopathy (modified from [8, 9, 51, 61])

Protein function	Gene (protein)	Presentation	Estimated prevalence in DCM (%)
Sarcomere	*ACTC* 1 (α-Cardiac actin)	HCM, NCCM, HCM	<1
	*MYH7* (β-Myosin heavy chain)	DCM, NCCM, HCM, myopathies	4–10
	*TNNT2* (Cardiac troponin)	DCM, NCCM, HCM	2–3
	*TPM1* (Tropomyosin)	DCM, NCCM, HCM	0.5–1.0
	*MYBPC3* (Myosin-binding protein C)	DCM, NCCM, HCM	2
Cytoskeleton	*TTN* (Titin)	DCM, NCCM, PPCM	12–25
Nuclear envelope	*LMNA* (Lamin A/C)	DCM +/− non-compaction phenotype, NCCM	4–6^a^
Nucleus	RBM20 (RNA-binding *protein*)	DCM	2–5
Ion channel	*SCN5A* (Sodium Channel, type 5)	Brugada, LQT3, AF, SSS, DCM	2

*DCM* dilated cardiomyopathy, *NCCM* non-compaction cardiomyopathy, *HCM* hypertrophic cardiomyopathy, *LQT 3* long QT syndrome 3, *PPCM* peripartum cardiomyopathy,* SSS* sick sinus syndrome, *AF* atrial fibrillation

^a^Up to 30%, if conduction disease is also present

### Titin

Titin (*TTN*) is the largest human protein and part of the sarcomere. Truncating TTN mutations are a common cause of non-ischemic DCM [38]. Moreover, TTN variants can also be found in approximately 1% of the general population, where they can be silent [39]. In PPCM, overlaps with genetic variants seen in DCM are described. In a large series of women with PPCM the distribution of truncating variants was similar to that found in DCM patients and these variants were the most prevalent genetic predisposition in both groups [33].

### Lamin A/C

Lamin A/C or LMNA is a protein encoded by the *LMNA* gene. Variants in that gene cause laminopathies affecting different types of tissues and organ systems—cardiomyopathy is the most prevalent laminopathy [40]. In approximately 6% of patients with DCM, LMNA variants were observed [41]. Mutations are often associated with atrial fibrillation, sinus node- or AV-node dysfunction, and ventricular arrhythmias, which can be present before left ventricular dilatation [8]. The LMNA variants were associated with the highest rate of heart transplantation [42].

### RNA-binding motif-20

Mutations in RNA-binding motif-20 (RBM20), a RNA-binding protein gene, can lead to DCM and are associated with high mortality [43]. In a review of 48 studies, carriers of the RBM20 mutation underwent transplantation at a younger age than patients with other variants [42].

### Sodium channel alpha unit

Mutations in sodium channel alpha unit (*SCN5A*) are usually associated with an increased risk of arrhythmias. Heterozygous dominant mutations are associated with syndromes such as long QT and Brugada syndrome, whereas some mutations lead to familial DCM [8].

### Sarcomere genes

About 10% of DCM cases could be assigned to sarcomeric genes (myosin-binding protein C [*MYBPC3*], myosin heavy chain [*MYH7*], troponin T [*TNNT2*], and troponin I [*TNNI3*]) in a meta-analysis involving more than 8000 patients [42].

## Differential diagnosis

In some patients the apex of the left ventricle shows a marked trabecularization. These cases must be distinguished from an important differential diagnosis, especially because there is an overlap not only in phenotype but also in genotype (Table 2).

### Left ventricular non-compaction cardiomyopathy

In the beginning of the twentieth century, a spongy appearance of the myocardium was described for the first time. Later this special morphology received more attention [44]. Imaging shows a characteristic appearance in left ventricular non-compaction cardiomyopathy (LVNC/NCCM). Bilayered myocardium with prominent trabeculations can be seen in children with LVNC as a result of arrest in compaction during embryonic development [45]. In adults, the disease must be divided into different subgroups. These range from a benign phenotype and normal ejection fraction to dilatative or hypertrophic manifestations, some of which may affect not only the left but also the right ventricle [46, 47]. Approximately 5% of all cardiomyopathies in children are NCCM. The prevalence of the disease in adults varies greatly—also depending on the imaging used [45]. Therefore, more than one imaging method should be used to confirm or rule out NCCM [45].

In most cases, however, echocardiography is the first-line diagnostic tool. Criteria for LVNC are deep intertrabecular recesses found mainly in the apex and the upper wall sections of the lateral and inferior wall, a two-layer wall structure, with an end-systolic ratio of >2 between the non-compact subendocardial layer and the compact subepicardial layer [48]. Imaging with CMR shows the extent of myocardial involvement, and with use of late gadolinium enhancement the degree of fibrosis [49].

Non-compaction cardiomyopathy can occur in isolation or in combination with various congenital heart diseases (e.g., ventricular septal defect, atrial septal defect, or pulmonary stenosis). Many gene loci have been identified that describe a combination of NCCM and other congenital heart diseases, such as hypoplastic left heart syndrome (Dystrobrevin alpha) or Ebstein anomaly (MYH7) [50]. Genetic variants that can be found code for sarcomeric proteins, nuclear envelope and Z‑band components, as well as sarcolemma protein and ion channels [51]. Genetic testing should be performed where possible in diseases associated with syndromes (I) but is also recommended for those without syndromic disease (IIA; [52]).

Non-compaction cardiomyopathy leads to an increased risk of arrhythmia and thromboembolic events [53]. While the diagnosis of NCCM does not justify the indication for anticoagulation in general, anticoagulation is recommended if atrial fibrillation or thrombus are present [54]. Various arrhythmias are associated with NCCM depending on the age of the patients. Bradycardias, Wolf–Parkinson–White syndrome, and atrioventricular block are more common in children whereas ventricular tachycardias, QT prolongation, and atrial fibrillation are more common in adults [55]. Holter-ECG is therefore recommended as part of the regular check-ups for these patients.

## Familial screening

The age at which DCM and NCCM occur varies also within families. Therefore, all first-generation relatives should receive a screening examination using ECG and echocardiography, being aware that an inconspicuous screening alone does not exclude the later occurrence of the disease [56]. Patients should therefore be cared for in centers to ensure individual therapy planning for the index patient and risk assessment for the rest of the family. This also enables more targeted decisions on the indication for genetic diagnostics.

In DCM this is recommended for patients with a familial disease and/or an accompanying conduction disorder (IIa). Patients with a “sporadic DCM” can be tested in individual cases (IIb; [52]). A clinical screening is also recommended, which should be carried out between the age of 10 and 20 years every 1–3 years and thereafter until the 60th year of life every 2–5 years (Fig. 1) [57].

## Therapy

In most cases, treatment of DCM means heart failure therapy based on drug treatment with angiotensin-converting enzyme inhibitors (angiotensin receptor blocker or angiotensin-receptor-/neprilysin-inhibitor if applicable), beta-blockers, and mineralocorticoid-receptor antagonists according to the current heart failure guidelines [16, 58]. Specific underlying causes should be treated specifically. Potentially harmful substances should be discontinued. Device therapy follows the guidelines. Some of the mutations in DCM can lead to arrhythmias and thereby to an implantation of an ICD despite an LVEF of >35%. If cardiac arrhythmia occurs, especially in NCCM, ablation treatments can be used to prevent further deterioration [59].

The treatment of patients with PPCM is a particular challenge. The administration of bromocriptine in PPCM has shown benefit in individual studies in addition to standard heart failure therapy [32]. However, there is no consensus about using bromocriptine, which may be considered in patients with PPCM especially if heart failure is moderate–severe (IIb; [29]).

## Conclusion

Dilated cardiomyopathy is one of the most frequent and important causes of heart failure, characterized by reduced systolic left ventricular function and dilatation of the left or both ventricles. The definition of underlying causes is still a challenge. The pathogenetic reasons are manifold including cardiotoxic compounds, metabolic, rheumatologic, and endocrinologic diseases, as well as infiltrative and infectious causes. Genetic aberrations have increasingly been defined as disease-causing in recent years. Genetic aberrations are associated with an increased risk for arrhythmias. Better diagnostic methods, the evolving field of biomarkers, and more sophisticated genetic analysis will probably allow for the development of therapy from a non-specific systolic heart failure treatment of non-ischemic cardiomyopathy to an individualized and cause-related treatment of specific cardiomyopathies in the near future.
